# CRISPR-Cas: A robust technology for enhancing consumer-preferred commercial traits in crops

**DOI:** 10.3389/fpls.2023.1122940

**Published:** 2023-02-07

**Authors:** Vipasha Verma, Akhil Kumar, Mahinder Partap, Meenakshi Thakur, Bhavya Bhargava

**Affiliations:** ^1^ Floriculture Laboratory, Agrotechnology Division, Council of Scientific and Industrial Research (CSIR) –Institute of Himalayan Bioresource Technology (IHBT), Palampur, India; ^2^ Academy of Scientific and Innovative Research (AcSIR), Ghaziabad, Uttar Pradesh, India

**Keywords:** commercial traits, CRISPR-Cas based genome editing, gene delivery, plant biotechnology, targeted mutation

## Abstract

The acceptance of new crop varieties by consumers is contingent on the presence of consumer-preferred traits, which include sensory attributes, nutritional value, industrial products and bioactive compounds production. Recent developments in genome editing technologies provide novel insight to identify gene functions and improve the various qualitative and quantitative traits of commercial importance in plants. Various conventional as well as advanced gene-mutagenesis techniques such as physical and chemical mutagenesis, CRISPR-Cas9, Cas12 and base editors are used for the trait improvement in crops. To meet consumer demand, breakthrough biotechnologies, especially CRISPR-Cas have received a fair share of scientific and industrial interest, particularly in plant genome editing. CRISPR-Cas is a versatile tool that can be used to knock out, replace and knock-in the desired gene fragments at targeted locations in the genome, resulting in heritable mutations of interest. This review highlights the existing literature and recent developments in CRISPR-Cas technologies (base editing, prime editing, multiplex gene editing, epigenome editing, gene delivery methods) for reliable and precise gene editing in plants. This review also discusses the potential of gene editing exhibited in crops for the improvement of consumer-demanded traits such as higher nutritional value, colour, texture, aroma/flavour, and production of industrial products such as biofuel, fibre, rubber and pharmaceuticals. In addition, the bottlenecks and challenges associated with gene editing system, such as off targeting, ploidy level and the ability to edit organelle genome have also been discussed.

## 1 Introduction

The goal of crop improvement is to achieve both quantitative and qualitative gains such as enhancing crop yield while simultaneously increasing the stress resistance, as well as the crop’s quality. Several decades of advancements in agricultural technology have resulted in a significant increase in crop productivity. But in the recent years, scientists and breeders have likewise steadily changed their emphasis to customer’s preference, as with the upliftment of living standard of consumers they begin to demand produce of higher quality. Consumers have demonstrated that crops with appealing appearance, texture, flavour, and aroma will have greater marketability. Today’s consumers emphasize on plants that not only have novel sensory attributes, but also have a high nutritional value, able to produce industrial products, and offer therapeutic components. Various procedures, including conventional cross breeding, chemical/radiation-mediated mutation breeding, molecular marker-assisted breeding, and genetic engineering have been effectively employed to improve various such crop attributes. As trait improvement and harnessing genetic variation is essential to every crop improvement programme. The advent of transgenic or genetically modified (GM) crops has expedited the crop improvement by resolving the major challenges (Raman, 2017). Because GM crops include the crops include the integration of foreign DNA into plant genomes, the benefits of this technology are being neglected by public outcry over a small number of mostly unfounded health and environmental concerns (Verma and Dwivedi, 2014; Bruetschy, 2019).

Site-specific nucleases (SSNs) based targeted genome-editing has evolved as a more sophisticated and precise method for manipulating the genome, with the ability to overcome these problems (Abdallah et al., 2015). This potent strategy has attracted the attention of breeders who want to improve critical features by modifying the genome without introducing foreign genes. Several SSN-based genome editing systems have been developed to cleave genomic sequences, including Zinc finger nuclease (ZFN), transcription activator-like effector nucleases (TALENs), and the recently developed clustered regularly interspaced short palindromic repeats (CRISPR)/Cas-mediated RNA-guided DNA endonucleases (CRISPR-Cas9). For precision genome engineering, SSN-based tools function as molecular scissors that may introduce DNA double-strand breaks (DSBs) and then activate diverse DNA repair processes, including non-homologous end-joining and homologous recombination (Ahmad et al., 2021). Genome editing with SSNs may provide a wide range of desirable genetic outcomes by exploiting DNA repair processes of DSBs. CRISPR-Cas9, a novel and incredibly useful family of SSNs, has been used to edit and regulate both DNA and RNA, enabling precise engineering of the plant genome (Hsu et al., 2013; Doudna and Charpentier, 2014; Zaidi et al., 2016; Aman et al., 2018; Mahas and Mahfouz, 2018; Bruetschy, 2019; Eid et al., 2021). As a multifunctional system, CRISPR-Cas9 based gene editing may be used to induce targeted heritable mutations by deleting, replacing, or inserting specified sequences in the genome precisely. This review offers a concise summary of the most recent genome editing methods for the precise modification of plant genomes together with a discussion of their applications, problems or concerns, and their usefulness in improving economically valuable relevant traits in crops. Point mutations are the most common mechanism through which major commercially viable quality features in crops are conferred (Huang and Gromiha, 2010). Thus, rather than random disruption of the gene, approaches that allow accurate and efficient base substitution in the target locus would considerably facilitate precise plant molecular breeding (Lu and Zhu, 2017). This innovative technology has capability to create new valuable traits in crops therefore boosting crop improvement programs (Cardi, 2016). Nutritional, sensory, medicinal, and industrial traits are important targets for crop improvement since they are highly valued by consumers. Thus, this article reviews the use of CRISPR-Cas technology in cereal, oilseed, and other horticulture crops with the goal of improving a wide range of quality traits (nutritional, sensory, and others) with an eye toward increasing their marketability. Improving traits preferred by consumers may require locating and modifying the genes responsible for regulating these processes. Several of these quality attributes are amenable to modification using CRISPR-Cas technology, that will result in greater customer satisfaction, subsequent purchases and market competiveness. To date, CRISPR-Cas9 has been used to edit the DNA of numerous crops, including soybean, potatoes, tomatoes, flax, rapeseed, camelina, cotton, chrysanthemums, and petunias, amongst others, to alter the consumer preferred traits that are commercially important (Tang et al., 2021). The use of CRISPR-Cas9 and other related technologies in plant genome engineering will not only hasten the progression of fundamental research, but it will also make the molecular breeding of crop plants easier making these technologies more promising for the upcoming future.

## 2 Genome editing tools for crop breeding

**Figure 1:**
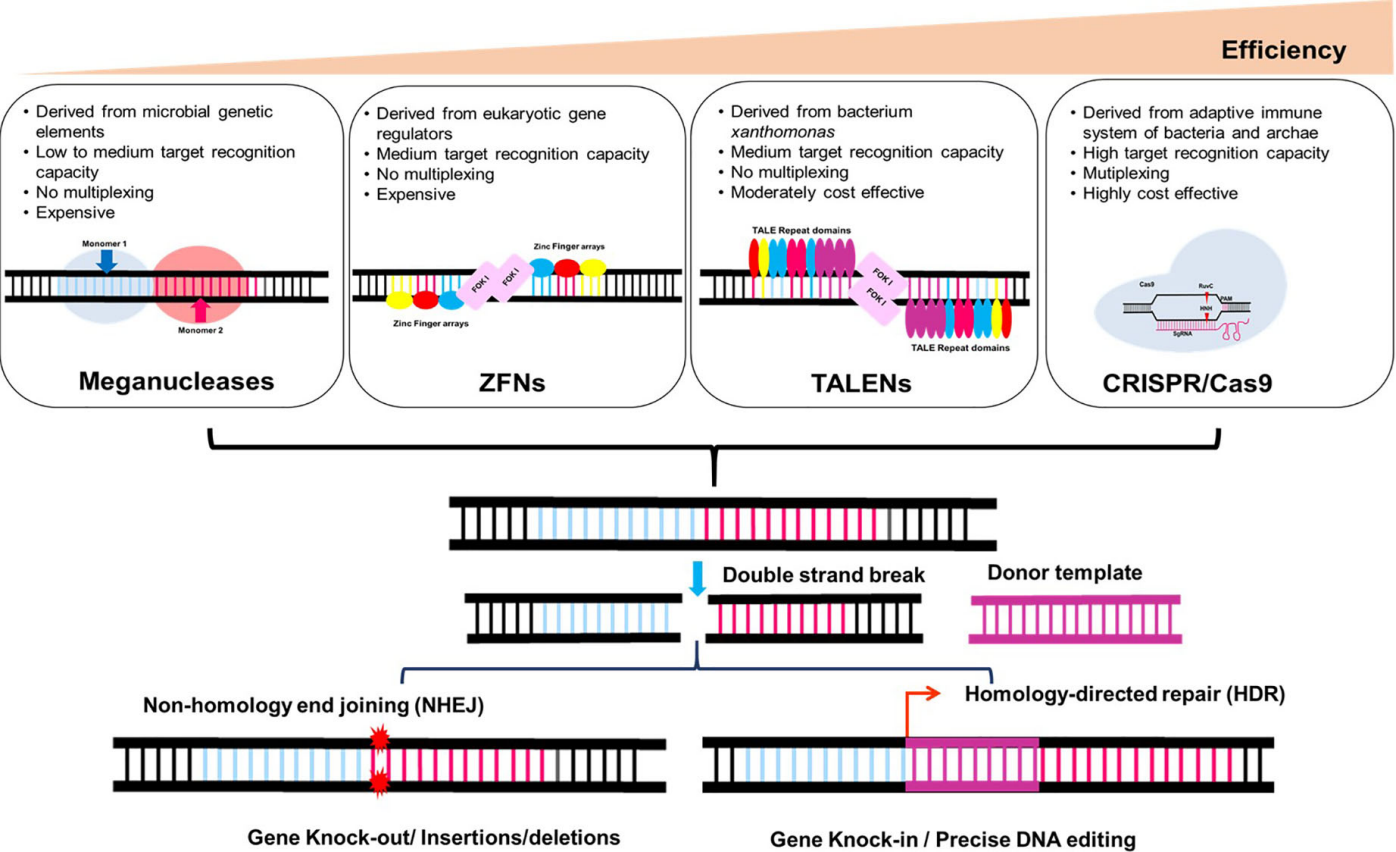
Plant genome editing in a targeted manner: Induction of double-stranded break’s (DSB) in DNA at a specific locus by engineered nucleases such as Meganucleases, ZFNs (Zinc finger nucleases), TALENs (Transcription activator-like effector nucleases) and CRISPR-Cas9 (CRISPR-Cas-mediated RNA-guided DNA endonucleases). Endogenous DNA repair mechanisms repair DSBs by non-homologous end joining or homology-guided repair.

Genome editing is currently one of the highly versatile and beneficial technologies available, with potential applications in the field of functional genomics as well as crop improvement. It is the set of sophisticated molecular biology methods that allow accurate, efficient, and targeted genomic locus modifications. The genome editing era has started with the discovery of first generation endonucleases, such as mega-nucleases (Epinat et al., 2003), ZFNs (Kim et al., 1996), and TALENs (Christian et al., 2010), which was followed by development and advancement of more sophisticated CRISPR-Cas technology (Jinek et al., 2012) to create site-specific DNA double-stranded breaks (DSBs). As first-generation genome-editing approaches, mega nucleases, ZFNs, and TALENs need rigorous procedures to attain target specificity. On the other hand, second-generation genome editing techniques like CRISPR-Cas9 employ simpler, quicker, and more affordable design and execution procedures. The CRISPR-Cas system’s simplicity, usability, and high efficiency have contributed to its emergence into the most widely utilised genome-editing tool **(** **Figure 1** **).**

### 2.1 Zinc finger nucleases (ZFNs)

ZFN was the first engineered nuclease that are widely employed to create DNA break resulting in the activation of DNA repair machinery (Petolino, 2015). ZFNs typically employed their “three fingers” to detect an 18 base pair target sequence in DNA. ZFNs are among the most powerful and flexible nucleases, with two domains: one for DNA binding and one for DNA cleavage. For DNA double-strand breakage, the type II restriction endonuclease FokI dimerizes to cleave the DNA strand (Smith et al., 2000). DSBs in DNA are repaired by two pathways: homologous recombination (HR) and non-homologous end-joining (NHEJ), resulting in gene mutations and gene knock-in or knock-down (Maeder et al., 2008; Foley et al., 2009; Lee et al., 2010). Research has shown that ZFNs with a larger number of zinc fingers (4, 5, and 6 fingers) are more specific and efficient at their intended targets. ZFNs have been used to produce fertile transgenic soybean plants using NHEJ mediated targeted insertions of multigene donors into an endogenous genomic locus (*FAD21a*) of embryogenic cells (Bonawitz et al., 2019). So far, ZFNs have been used to modify the genetic makeup of many different plant species, including arabidopsis, tomato, rice, apple, tobacco, petunia, soybean, maize, and fig (Gaj et al., 2013; Cantos et al., 2014; Hilioti et al., 2014; Martinez-Fortun et al., 2017; Ran et al., 2017). The principal drawbacks of ZFNs include labor-intensive, complex and expensive construction of protein domains for each specific locus target in the genome, as well as imprecise cleavage in the target sequence of the genome owing to erroneous protein domain interaction.

### 2.2 Transcription activator-like effector nucleases (TALENs)

TALENs, like ZFNs, are made up of transcriptional activator-like effector (TALE) repeats and the FokI restriction enzyme. Because each TALE repeat selectively targets a single nucleotide, TALE repeats may target more target sites than ZFNs, allowing for more versatile target design. Many plant species have successfully been altered their genomes using TALENs (Ran et al., 2017). TALENs mediated targeted mutagenesis of the Caffeic acid O-methyltransferase replaced with Caffeic acid O-methyltransferase (*COMT*) in sugarcane resulted in improved the cell wall composition for enhanced bioethanol production (Jung and Altpeter, 2016; Zaman et al., 2019). Similarly, multi-allelic mutagenesis of *COMT* mutants using TALENs resulted in the reduction of 19.7% lignin content and a decrease in syringyl to guaiacyl (S/G) ratio with improved saccharification efficiency. Such targeted alterations in the *COMT* gene increased sugar content in sugarcane without causing a loss of biomass (Kannan et al., 2018). By interrupting the genes for fatty acid desaturase (*FAD*) using TALENs, soybeans with high oleic acid and low linoleic acid contents were produced, suggesting that this technology may be utilised to alter the nutritional profiles of crops (Demorest et al., 2016). Similarly, the betaine aldehyde dehydrogenase (*BADH2*) gene has been disrupted using TALEN technology to make rice with fragrance (Shan et al., 2015). In order to create purple tomatoes with increased anthocyanin levels, a strong promoter was inserted upstream of the gene regulatinganthocyanin biosynthesis using TALENs (Čermák et al., 2015). These examples demonstrate the enormous potential of the TALEN as gene editing technology for enhancing commercially important agricultural traits. Likewise, ZFNs, this genome editing tool has a few drawbacks such as it is laborious, time consuming and have low mutation rate (Becker and Boch, 2021).

### 2.3 Clustered regularly interspaced short palindromic repeats-Cas9 (CRISPR-Cas9)

**Figure 2:**
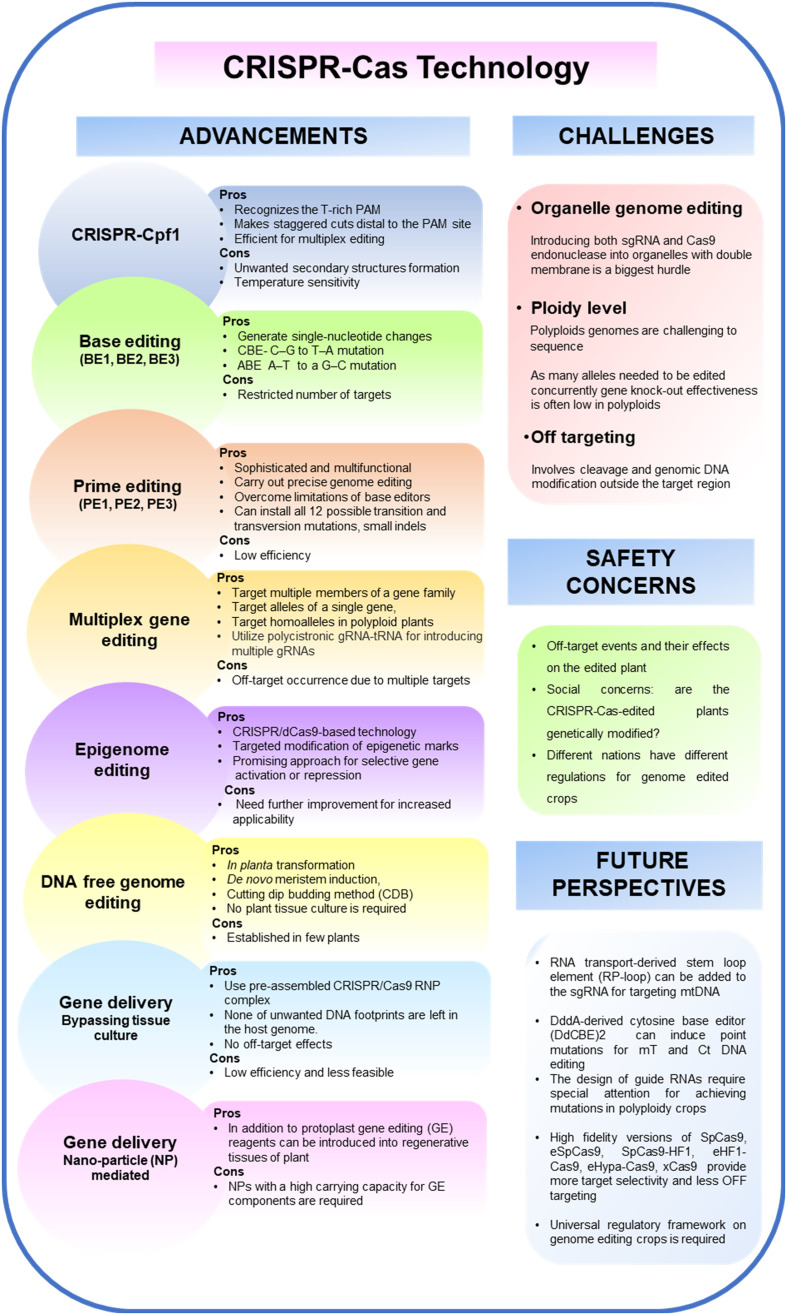
An overview of CRISPR-Cas technology for genome editing in plants: Advancements, challenges, concerns and future perspectives.

CRISPR-Cas based genome editing has sparked another revolution in targeted genome engineering. Unlike ZFNs and TALENs, CRISPR-Cas9 based genome editing is effective, economical, and effortless in generating DSBs in DNA. In addition, it facilitates the creation of any genomic target, multiplexing, simple prediction of off-target regions, and straightforward delivery. CRISPR-Cas9 system originated from *Streptococcus pyogenes* (Sp) is well conserved. Its discovery was a 20th-century breakthrough since it is a unique technology that is being explored by researchers from different fields such as bioinformatics, biotechnology, and microbiology (Miki et al., 2018). Due of its versatility, simplicity, efficacy, and wide range of applications, the CRISPR-Cas9 system is used in fundamental as well as applied research. CRISPR-Cas9 uses DNA–RNA interaction and requires a target-site-specific 18–20 bp oligonucleotide sgRNA (Single guide RNA) to edit genes. In case of SpCas9, designed gRNA binds to a 5′-NGG-3′ protospacer adjacent motif (PAM) sequence at the 3′ end of the target sequence Cas9 is often used to generate DSBs at the specified target region in the genome to cause mutations (Feng et al., 2018). Heritable genomic alterations and transgene-free plants were produced as a consequence of the targeted plant genome editing made possible by CRISPR-Cas9 (Hu and Gao, 2023). Utilizing the CRISPR-Cas system several crops have shown both qualitative and quantitative gains (Zhang et al., 2020; Carrijo et al., 2021; Confalonieri et al., 2021; Eid et al., 2021; Oz et al., 2021; Zhang et al., 2021; Biswas et al., 2022; Lu and Tian, 2022). An overview of CRISPR- Cas technological advancements are shown in **Figure 2**.

#### 2.3.1 Generation of gene knock out and gene knock-in

Depending upon, how intrusive foreign DNA reacts, the CRISPR-Cas9-based system’s mode of action can be categorised into three primary phases. The first phase is the procurement stage, which involves recognising foreign DNA and inserting a spacer sequence into the CRISPR array based on the target site in the DNA (Hahn et al., 2017). Second, the Cas9 protein is produced and the CRISPR array is translated into a precursor RNA transcript during the expression step (precrRNA). This precrRNA later combined with a non-coding trans-acting CRISPR RNA (crRNA) and Cas9 to generate mature crRNA (Shin et al., 2016). The last step of interference in which the crRNA directs Cas9 to cleave and destroy the specific target spot in DNA. In eukaryotes, DSBs may be repaired *via* either NHEJ or HR mechanisms (Wang et al., 2016). Due to unanticipated deletions or insertions in the target sequence, DSBs repaired by NHEJ are prone to errors that may result in gene knock-outs (**Figure 1**). HR can’t be anticipated, but a DNA donor template with homologous flanking regions that permits gene editing or knock-in is necessary for homology-directed repair (HDR). Plants rely mostly on the NHEJ-repair mechanism, which has been used extensively to achieve targeted KO of target genes. In general, when both the DSB repair pathways are equally available in an organism the frequency of NHEJ is higher as compared to the HDR (Vu et al., 2017). *GmFAD2-1A* and *GmFAD2-1B* gene knockdown in soybean using the CRISPR-Cas9 tool enhanced oleic acid concentration by up to 80%, while decreasing linoleic acid to 1.3-1.7% in the mutants (Do et al., 2019). In the case of hexaploid *Camelina sativa* knock out of three pairs of homologous *FAD2* gene results in increased monounsaturated fatty acids (MUFAs) in mutant lines (Lee et al., 2021). HDR is a highly preferable DSB repair pathway that results in accurate gene knock-in or substitution of genes. CRISPR-Cas9-mediated targeted site knock-in has been used to increase the precision, efficiency, and expression of the reporter gene to a specific target site (Kim et al., 2020). HDR-mediated gene substitution with 9% frequency was seen in tobacco protoplasts transiently expressing gRNA and Cas9 at the target *AvrII* site *NbPDS* gene utilising a DNA template (Li et al., 2019). Currently, site-directed insertion of a desired gene, promoter, or specific DNA fragment at a precise location using CRISPR-Cas9 is in requisition. For example, arabidopsis lines transformed by CRISPR-Cas9 along with DD45 promoter show increased knock-in efficiency in egg cells when compared with the other regenerative cells or tissues (Miki et al., 2018). In variety of crops including tomato, soybean, potato, tobacco and poplar, precise and heritable modifications have been made using HDR-mediated target site knock-in of a gene (Svitashev et al., 2015; Hegde et al., 2021; Movahedi et al., 2022). Although HDR mediated gene editing (or KI) has promise for crop breeding, but it is still more challenging to implement. In eukaryotic cells, its success rate is limited (a few percent or less), and this is linked to the technical challenge of delivering the donor template at the proper time and in close proximity to the DSB. According to Wang et al. (2014), NHEJ DNA repair mechanism can be accelerated by DNA insertion at the DSB site efficiently once a donor DNA template is exogenously introduced. To boost the incidence of targeted insertions *via* NHEJ, however, short homologous chromosomal segments are added to the terminal ends of donor DNA to generate compatible ends or microhomology with the DSB surrounding sequence (Dong et al., 2020). However, chemically stabilised double-stranded oligodeoxynucleotide (dsDNA) and 5’- phosphorylated ends may potentially be used to enhance target insertion through the NHEJ pathway (Lu et al., 2020). In the absence of NHEJ pathway, this repair mechanism occurs via micro homology mediated end joining (MMEJ) (Schmidt et al., 2020).

## 3 Advancements in genome editing

### 3.1 CRISPR-Cpf1

One of the major disadvantages of the CRISPR-SpCas9 gene editing system is the formation of off-target cleavage sites, that occurs as a consequence of the gRNA complexing with mismatched complementary target DNA within the genome. Several modifications have been made to the Cas9 enzyme to reduce off-targeting and increase target selectivity (Lee et al., 2019). CRISPR-CpfI is one of the orthologs of CRISPR-Cas9 discovered to enhance target selectivity. It was formerly known as Cas12a and is a *Prevotella* and *Francisella*-derived class II type V endonuclease (Zetsche et al., 2017). CRISPR-Cpf1 (Cas12a) from *Alicyclobacillus acidoterrestris* is a novel member of CRISPR-Cas system. Presently, AsCpf1, LbCpf1, and FnCpf1 are the most common types of Cpf1 used in genome editing. CpfI, in contrast to Cas9, requires just a single guide RNA (crRNA) to recognise its target, and it may target the A/T-rich area of the genome, expanding the number of sites it can change. Cas9, on the other hand, exclusively targets G-rich PAM sequences (Tang et al., 2016). Cas9 endonuclease cleaves the genome into blunt ends, while Cpf1 generates cohesive ends that are effective for gene insertions in plants (Yin et al., 2017). The CRISPR-Cpf1 system has endonuclease activity, and can induce DSBs, also have RNase III activity for pre-CRISPR RNA processing and doesn’t need tracrRNA. Furthermore, since a single crRNA array transcript may target several genomic locations, Cpf1 allows multiplexed genome editing. The CRISPR-Cpf1 system in monocot and dicot plants makes it easier to delete, insert, edit, and tag genes with fewer off-target effects. Recently, both model plants and crops, have exploited Cpf1s for crop genome editing (Endo et al., 2019; Wolter et al., 2019; Alok et al., 2020). The CRISPR-LbCpf1 system was used to edit the genome of allotetraploid cotton with an editing efficiency of 87% and no off-target effects were observed. CRISPR-Cpf1 in combination with the CRISPR-Cas9 system may activate the targeted gene knock-in mechanism for crop improvement (Kaul et al., 2020). Cpf1 has constraints, such as a shorter crRNA and specific temperature requirements for genetic transformation in plants (Lee et al., 2019; Schmidt et al, 2020). The shorter length of Cpf1 crRNAs compared to Cas9 may result in the formation of unwanted secondary structures and decreased efficiency, as shown in maize (Malzahn et al., 2019). The temperature sensitivity of Cpf1-mediated genome alteration in plants is a major disadvantage. The effects of low temperature on the activity of AsCpf1, FnCpf1, and LbCpf1 have been found in arabidopsis, rice, and maize (Kumar et al., 2020).

### 3.2 Base editing

**Figure 3:**
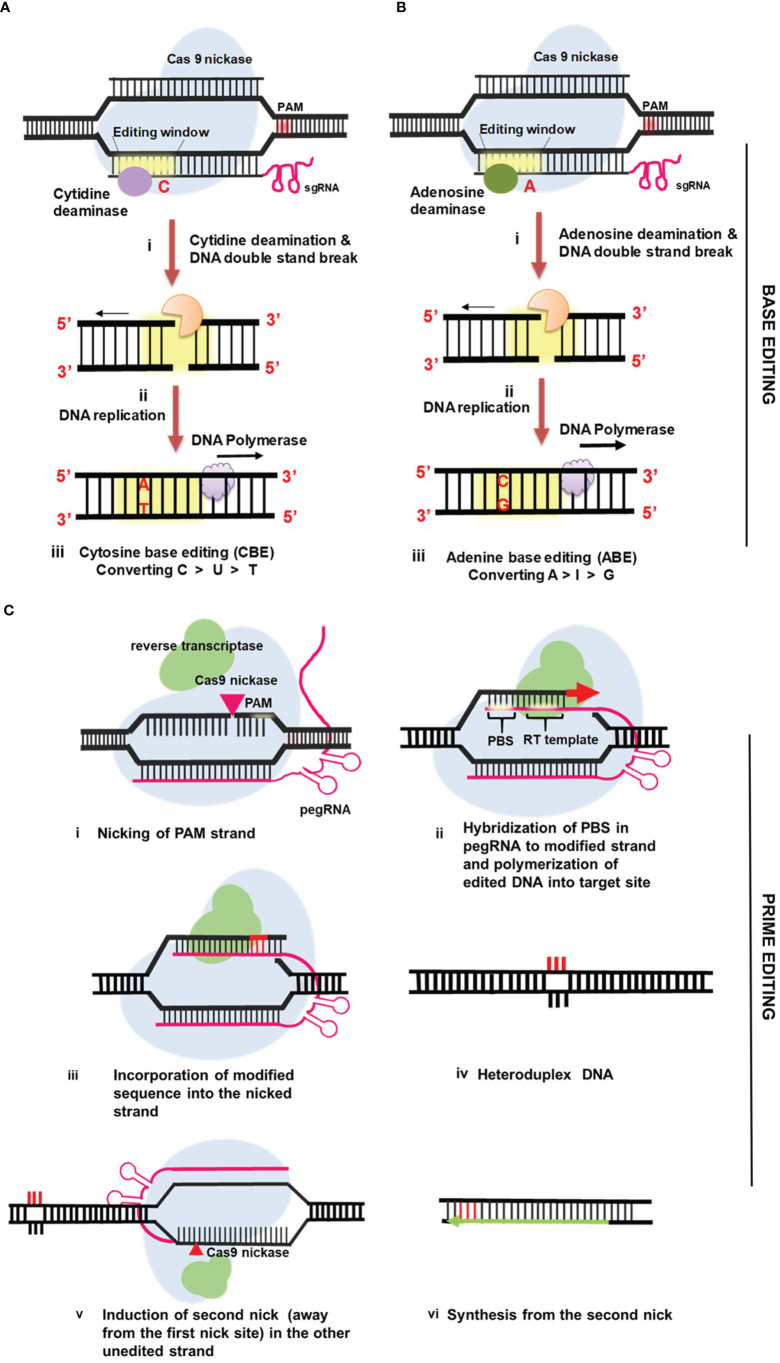
Mechanism of precise genome editing in plants using base editors and prime editors **(A)** Cytosine Base Editing (CBE) **(B)** Adenine Base Editing (ABE) **(C)** Prime Editing (PE).

The CRISPR-Cas9 system can be used to knock out or insert new genes, however this method cannot change the sequence of DNA at the base level. Because of these restrictions, different methods are needed to achieve stable and accurate genome editing in plants. Recently, a breakthrough method known as “base editing” has evolved that permits exact nucleotide changes in a programmed way without causing gene disruption or necessitating a donor template (Komor et al., 2016). DNA base editors, which are generally consisting of a catalytically inactive nuclease linked to a base-modifying catalytically active enzyme, are used in base editing systems **(** **Figures 3A, B** **).** Currently, two DNA base editors are in use: the cytidine base editor (CBE) and the adenine base editor (ABE). Cytosine (C) is deaminated using CBE to produce uracil (U). During DNA replication, uracil (U) is read as thymine (T). As a result, CBE causes a single base shift from C:G to T:A (Komor et al., 2016). The inactive CRISPR-Cas9 domain in ABE is linked to adenosine deaminase, which helps to convert adenine (A) to inosine, which is read as guanine (G) during DNA replication **(** **Figure 3B** **).** As a result, ABE causes A:T to G:C base substitutions (Nishida et al., 2016). The first generation base editor was created by combining cytidine deaminase with Cas9 nickase (nCas9) (Komor et al., 2016). This base editing system comprises of a single-guide RNA with nCas9 to target the site and deamination on the non-complementary strand, as well as a single break in the target DNA strand to initiate the conversion of G to A on the opposite strand through DNA replication (Kumlehn et al., 2018). The base excision repair activity eliminates uracil *via* uracil N-glycosylases (UNGs). As a result of this, the BE1 technique is inefficient for altering single bases *in vivo* (Komor et al., 2016). Furthermore, the restricted number of targets is constrained by the short base editing window and the necessity of certain PAM sequences. Given these limits, as well as its low efficiency, a second-generation base editor (BE2), was created as an improved version of base editors (Wang et al., 2017). BE2, a novel base editing tool was created by using uracil DNA glycosylase to disrupt the excision repair process and boost editing efficiency by thrice. Moreover, BE2 produces very few indels (0.1%) during base editing, making it a great choice in instances where indels are unwanted (Komor et al., 2016). BE3 is a third-generation editor composed of rat cytidine deaminase APOBEC1 linked to a Cas9 nickase [nCas9 (D10A)] and the UGI. BE3 is required for the conversion of targeted cytidine to thymidine in DNA, and its PAM efficiency and specificity have been improved (Hess et al., 2016). Unlike cytidine deaminases, adenine DNA deaminases do not occur in nature. Significant protein engineering and directed evolution were used to construct ABEs using *Escherichia coli TadA* (Liu et al., 2017). *TadA* is a tRNA adenine deaminase from *E. coli* that converts adenine to inosine in the anticodon loop of tRNA Arg. It is similar to APOBEC. The first ABEs were created by fusing a *TadA* with a catalytically defective CRISPR-Cas9 mutant (Gaudelli et al., 2017). ABE7.7, ABE7.8, and ABE7.9 are the most active and sequence-compatible ABEs. The seventh-generation ABEs (ABE7.10) were proposed for efficient and pure A:T to G:C conversion in a wide range of targets. ABEs easily generate point mutations and programme all four transitions (C to T, A to G, T to C, and G to A), significantly increasing base editing. SNPs and minor indels cause most agronomically relevant features which BEs can repair for precision crop breeding (Zhao et al., 2011; Maccaferri et al., 2015; Wu et al., 2021). Previous research has proved the efficient use of base editors (ABE or CBE) in a vast array of plant species (Shimatani et al., 2017 and Zong et al., 2017; Kang et al., 2018; Li et al., 2018; Tian et al., 2018; Yan et al., 2018; Zong et al., 2018; Endo et al., 2019).

### 3.3 Prime editing

Prime editing is a unique, sophisticated, multifunctional, and precise genome editing method that generates all potential transition and transversion mutations as well as small indels in the DNA (Anzalone et al., 2019). Using novel prime editing method genetic information at the target loci has been successfully incorporated into the genome without DSBs. This approach employs the single-guided RNA referred known as primary editing gRNA (pegRNA) and the fusing of reverse transcriptase to the C-terminal of a Cas9 H840A nickase. The pegRNA may be utilized as a reverse transcription template (Vu et al., 2020). It is a modified sgRNA that incorporates a primer binding site (PBS) and the genomic sequence to be copied at its 3’ end **(** **Figure 3C** **).** Reverse transcriptase uses the nicked strand-free 3’OH group as a template to transcribe the genetic material from the pegRNA 3’ end extension (Vu et al., 2020) Nucleotide substitutions during DNA repair may occur if pegRNA were synthesized using modified nucleotides. Prime editing does not require the donor DNA during homologous DNA repair (HDR) process. Prime editor 1 (PE1), one of three basic editing techniques, has a Cas9H840A nickase with a wild-type Moloney murine leukaemia virus RT fusion at the C-terminus (M-MLV-RT) (Zhan et al., 2021). Prime Editor 2 now includes the proposed M-MLV-RT pentamutant (D200N/L603W/T330P/T306K/W313F), which has improved thermostability, processivity, DNA-RNA substrate affinity, and inhibited RNase H activity (PE2). In comparison to PE1, this system improved point mutations by 1.6 to 5.1-fold and consistently generated more efficient targeted insertions and deletions. In the PE3 method, two paired DNA strands are double-nicked, which could result in a DSB and indel creation as a result of NHEJ repair. Several parameters, including reverse transcriptase type, temperature, template length, PBS length, and the necessity for a second nick, may influence prime editing efficiency in plants. Prime editors have been shown to be quite helpful for both basic biological research and crop breeding. The upgraded prime editors have much lower off-target frequency in plants than the base editors (Jin et al., 2019). Multiple independent studies have shown that PE has a poor effectiveness for modifying the genome of plants (Xu et al., 2019; Butt et al., 2020; Jiang et al., 2020; Lin et al., 2020; Lu et al., 2020; Tang et al., 2021). These experiments demonstrate that PE has the potential to become a viable method for precise genome editing in crops, but more work has to be done to increase the efficiency of prime editing before this technology can be used more widely. PEs may execute targeted saturation mutagenesis to create beneficial genetic variants with increased agronomic performance for crop genes lacking functional SNPs (Molla et al., 2021).

### 3.4 Epigenome editing

One of the primary targets of heritable alterations in gene expression and cellular function is epigenetic modification. Targeted gene expression control was made possible by methylating DNA, creating non-coding RNAs and modifying histones (Kumar and Mohapatra, 2021). Addition of a methyl group to the C’5 position of cytosine residues is the hallmark of DNA methylation, a well-known epigenetic change associated with gene silencing. Histone modifications include acetylation, methylation, ubiquitination, and phosphorylation of amino acid residues in the N-terminal tail (Zhan et al., 2021). There are enzymes (writers) that catalyse the addition of epigenetic markers, such as DNA methyltransferases (DNMTs), histone acetyltransferases (HATs), and histone lysine methyltransferases (HKMTs), and enzymes (erasers) that catalyse their removal, such as DNA demethylases, histone deacetylases (HDACs), and histone demethylases (Zhang et al., 2021). To dynamically modulate epigenetic modifications, which in turn affects chromatin conformation and transcriptional regulation, writers and erasers must interact (Jaenisch and Bird, 2003; Torres and Fujimori, 2015; Feinberg et al., 2016). However, in very rare circumstances, epigenetic changes may cause a permanent change in gene expression without affecting the genome sequence. These epimutations are called epigenetic alterations. Transgenerational inheritance of changes in DNA methylation is the primary source of the matching epigenetic allele, often known as a “epiallele” (Hauser et al., 2011; Quadrana and Colot, 2016; Lloyd and Lister, 2022). Floral organ development (Jacobsen and Meyerowitz, 1997), flowering time (Soppe et al., 2000), starch metabolism (Silveira et al., 2013), fruit ripening (Manning et al., 2006), vitamin E accumulation (Quadrana et al., 2014), and sex determination are just some of the many developmental/physiological processes that have been shown to be regulated by epialleles in plants (Martin et al., 2009). The ability to regulate transcription without changing DNA sequences has led to the development of epigenome editing technologies. Epigenome editing, the targeted modulation of epigenetics at a single gene, is often accomplished by fusing a specific epigenetic modifier with a targeting module of the standard genome editing system. The catalytic domain of an epigenetic modifier may be linked to programmable DNA-binding modules like ZF, TALE, and catalytically inactive Cas9 (dCas9). Epigenome editing has great promise as a means of controlling gene expression in a targeted manner. Epigenetic alteration in plants may lead to the generation of new epialleles linked to desirable features for crop development. Several reports have been made on investigations of epigenetic alterations in plants that are specifically targeted (Gallego-Bartolomé et al, 2018; Lee et al., 2019; de Melo et al., 2020). An effective technique for crop improvement might be epimutations associated with desirable features. Understanding the fundamentals of how epigenetics influences yield, quality, disease resistance, and stress tolerance that are vital to farmers and industry necessitates the research of epimutations across a broad variety of crop species. Epigenome editing will help future crops to adapt with harsh environmental circumstances. Earlier it has been demonstrated that the conserved histone variant (H2A.Z) is required for transcriptional control, defensive responses, and plant growth, development, and flowering. Using CRISPR-Cas9 mediated approach in tomato, *Slhta9, Slhta11* double-mutant and *Slh2a.z* were created and discovered that these mutations reduced the fresh weight of tomato fruits. mRNA-seq data demonstrated that genes *SlPSY1, SlPDS*, and *SlVDE*, which encode important enzymes in the production pathway of carotenoids, were considerably elevated in later ripening stages, commensurate with the enhanced carotenoids in *slh2a.z* double-mutant fruits. Previous research has reported that *Slh2a.z* regulates carotenoids and offers a resource for studying *Slh2a.z*-dependent gene expression (Yang et al., 2021b). While, epigenome engineering has the potential to be a game-changing tool for the precise regulation of epigenetic markers, further research is required to fully realise its potential.

### 3.5 Multiplex gene editing

In multiplex genome editing, many targets, both related and unrelated, can be simultaneously edited. This feature of CRISPR-Cas provides several applications such as targeting multiple members of a gene family, simultaneous targeting of many closely related sequences, numerous alleles of a single gene and homoalleles in polyploid plants (Li et al., 2013). Several gRNAs were inserted as polycistronic transcripts or separate expression cassettes with their own promoters, and they were processed into mature gRNAs by either endogenous or exogenous nucleases, the CRISPR-Cas9 system makes the latter approach very effortless. The most prevalent method for the generation of numerous gRNAs is to express each individual gRNA using its own promoter and terminator, a strategy that may be implemented using either distinct plasmids for each gRNA or by connecting several cassettes on the same vector (Xing et al., 2014; Ma et al., 2015). Nonetheless, many studies have reported the delivery of gRNAs and Cas9 as ribonucleoprotein complexes, and this strategy might potentially be utilized to carry multiple gRNAs (Woo et al., 2015; Liang et al., 2017). In order to generate many gRNAs from a single transcript, which is then processed to generate the mature gRNAs, a number of alternative techniques have been devised. Like CRISPR, the Cys4 system employs a bacterial endonuclease to edit DNA. Here, several gRNAs are placed in tandem, interspersed with Cys4 recognition sites; this allows for post-transcriptional release of mature gRNAs upon coexpression of Cys4 (Cermak et al, 2015). The CRSIPR/Cpf1 approach is an option that has the benefit of using the same enzyme (Cpf1) to separate the polycistronic gRNAs and to target the genomic loci for alteration (Wang et al., 2018). Thirdly, self-cleaving ribozymes may be employed to liberate an internal gRNA from a ribozyme-gRNA-ribozyme complex (Tang et al., 2016). Compare to this, the tRNA-dependent gRNA method has been used in many studies for multiplex genome editing in plants. All organisms manufacture tRNA as a longer precursor, which is then digested by endogenous RNases to form the mature tRNA. The creation of progRNA arrays with target sites for tRNA maturases allows the automated release of individual gRNAs (Hashimoto et al., 2018). The first emphasis of multiplex genome editing in plants was on input traits like herbicide resistance, and more over a hundred simultaneous targeting events have been documented. Since then, this discipline has grown to incorporate plant growth, metabolic engineering, hormone sensing and biosynthesis, among other significant characteristics. The genes *OsGSTU, OsMRP15*, and *OsAnP*, which are involved in anthocyanin transport and accumulation, were concurrently changed in a rice line with purple leaves to produce mutants with green leaves. The *OsWaxy* gene, which makes granule-bound starch synthase, is also targeted by the same researchers. They recovered plants with mutations in one or two but not all three loci, and the mutants’ amylose content dropped from 14.6% to 2.6% (Ma et al., 2015). Six gRNAs were used to target five genes in the tomato carotenoid biosynthesis pathway, with two targets in the *SGR1* gene and one target each in the genes *LCY-E, Blc, LCY-B1* and *LCY-B2*. By promoting the formation of lycopene and preventing its conversion to β- and α-carotene, the lycopene content of tomato fruits was intended to be increased (Li et al., 2018)

### 3.6 DNA free genome editing

The primary objective of plant gene editing is to create alterations to the genome that are stable, heritable, and nonmosaic, allowing the resultant trait or phenotype to be consistently preserved and passed on to future generations (Huang et al., 2022). Thus, when the intended gene editing has been performed, all traces of the gene-editing components must be eliminated (Metje-Sprink et al., 2019; Miroshnichenko et al., 2019). Recent advancements in genome editing tools including DNA-free delivery methods and base editing systems solved this problem and provide a wide opportunity to edit plant genomes in a precise manner (Bhattacharjee et al., 2020). Advanced tools such as direct delivery method, and delivery of *in vitro* assembled ribonucleoprotein (Cas9/gRNA) and use of virus-like particles and employment of bacterial secretory systems for Cas/gRNA delivery are the main approaches that have been employed to accomplish DNA-free genome editing. Such complex formulations can be delivered into plant cell by agrobacterium mediated approach, protoplast transformation, microinjection, electroporation, particle bombardment and liposomes mediated transformation (Zhang et al., 2017). Transient transfection may be used to directly insert CRISPR ribonucleoprotein (RNP) or plasmids with the Cas and sgRNA sequences into protoplasts, enabling the regeneration of recombinant DNA-free plants without raising GMO concerns (Hsu et al., 2021). Furthermore, in order to attain DNA free genome editing, CRISPR-Cas9 RNPs were employed for targeting two endogenous genes (*FRI* and *PDS*) in cabbage and Chinese cabbage protoplasts. As a result of this, local insertion and deletion mutations (indels) were obtained (Murovec et al., 2018). To enable DNA-free genome editing in canola, RNPs (composed of LbCas12a and a single guide RNA) were transformed using polyethylene glycol (PEG) with a mutation frequency of 40% protoplast derived plants (Sidorov et al., 2022). Although, DNA-free genome editing using protoplast is simple, efficient, and transferable in a generation but in some monocot crops, the protoplast isolation and regeneration of shoots remain a bottleneck. Besides this particle bombardment mediated DNA-free genome editing is restricted to some species due to resulting in cells rupturing, therefore, the plant transformation method needs to be optimized for achieving success in this system.

### 3.7 Advances in the delivery of gene-editing reagents into plant cells

The dependency on plant genetic transformation and regeneration processes is a big barrier for many species when it comes to gene editing. The most prevalent method of gene delivery is agrobacterium-mediated transformation, which involves integrating the DNA to be transferred into its transfer DNA (T-DNA), which is then integrated into the plant genome. Particle bombardment using a gene gun is another method used in monocot species. Both procedures result in the random integration of DNA into plant genomes (Laforest and Nadakuduti, 2022). Foreign DNA insertion into host DNA is deemed genetically modified and needs regulatory oversight. Plant cell walls, pose a specific barrier to the introduction of gene-editing reagents. Non-transgenic genome editing is possible in protoplasts, which, like animal cells, lack cell walls. Protoplast transfection using plasmids containing gene-editing agents or RNPs has resulted in the regeneration of complete plants in a few species (González et al., 2020; Laforest and Nadakuduti, 2022). However, complete regeneration of plant from single-celled protoplasts, necessitates extensive tissue-culture procedures that result in frequent and undesirable somaclonal variation. A recent study of protoplast regenerants identified anomalies in chromosome structure and number that might impact plant phenotype (Fossi et al., 2019). As a result, innovative ways for overcoming these issues, particularly those that do not rely on tissue culture, seems useful (Ji et al., 2020) and are discussed below.

#### 3.7.1 Gene delivery bypassing plant tissue culture

##### 3.7.1.1 *In planta* transformation

**Figure 4:**
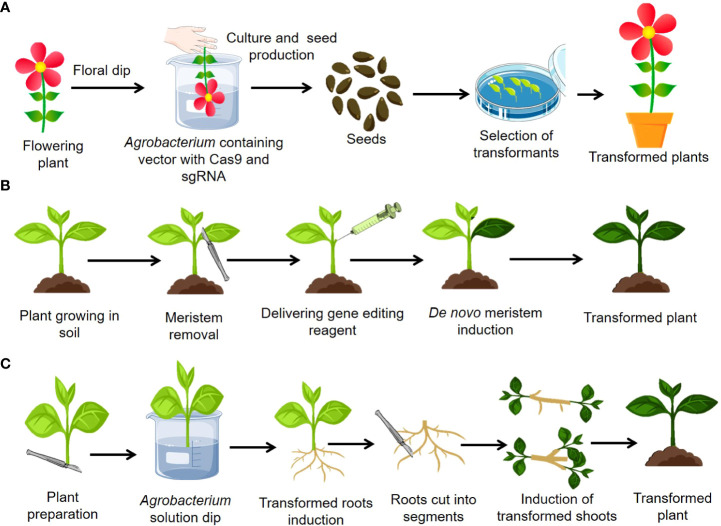
Advances in delivery of gene editing reagents in plants bypassing tissue culture **(A)** *In planta* transformation **(B)** *De novo* meristem induction method **(C)** Cut Dip Budding (CDB).

Numerous ways for plant genetic transformation that do not depend on *in vitro* regeneration have been developed. Among such methods, floral dip method or “*in planta*” transformation is well known **(** **Figure 4A** **)**. The stage of a plant is essential for its appropriate floral transformation. Floral-dip mediated transformation of CRISPR-Cas9 components is straightforward and economical but require use of efficient promoters for better editing efficiency (Zlobin et al., 2020). Various constitutive (*CaMV35S* and arabidopsis *UBI10*) and germline promoters such as *MGE1, YAO, RPS5a, AG*, and *ICU2* have been found efficient for creating mutants efficiently in plants (Yan et al., 2015; Castel et al., 2019). Using CRISPR-Cas9, the egg cell-specific promoters *EC1.2* and *EC1.2::EC1.1* have shown comparable editing efficacy in Arabidopsis (Wang et al., 2015; Feng et al., 2018; Zeng et al., 2020). Until yet, floral dip-mediated genome editing has only been used in arabidopsis (Wang et al., 2015; Castel et al., 2019). However, this method was used to successfully genetically transform the other plant species such as flax, tomato, radish, brassica, and wheat (Zale et al., 2009; Sharada et al., 2017). With the use of the donor template vector and the sequential floral dip approach, CRISPR may be used to knock in genes into germline cells or other regenerative cells. In this case, a donor template consisting of the left and right homology arms is required. For instance, one of the donor vectors consists of two T-MLO homology arms and a GFP coding region. This GFP donor vector was inserted with the CRISPR-Cas9 vector into wheat protoplast for GFP knock-in (Wang et al., 2014). The arabidopsis Cas9 line was used for sequential floral-dip transformation using germline-specific promoters including *DD45, Lat52, YAO*, and *CDC45*. Cas9 controlled by the *DD45* promoter was shown to be more effective for knock-in in egg cells and early embryos than in other regeneration organs (Miki et al., 2018). The transformation efficiency of flax was found to be between 50 and 60%, which is comparatively higher than that of arabidopsis using the floral-dip method of gene transfer (Bastaki and Cullis, 2014). The primary advantage of this delivery strategy was that no plant tissue culture facility was required. This is the most common and widespread method for altering the arabidopsis genome worldwide. The drawback of floral-dip-mediated CRISPR-Cas9 component delivery is that it is restricted to a small number of plants, including arabidopsis, flax, and tomato, and is less effective due to limited flower and seed production.

##### 3.7.1.2 *De novo* meristem induction

The root apical and shoot apical meristems are responsible for plant growth. The continual actions of these meristems, which are started and sustained by Development Regulators (DRs), give rise to a variety of cell types, resulting in the formation of shoots and roots. As previously reported (Ckurshumova et al., 2014), ectopic expression of DRs such as *BABYBOOM (BBM)*, *WUSCHEL2 (Wus2)* and *SHOOT MERISTEMLESS (STM) MONOPTEROS (MP)* might cause the creation of meristem-like structures. Recently developed, gene editing method has simplified or eliminated tissue culture by reprogramming genome-edited somatic cells into meristems *via* co-expressing DRs and gene editing components, allowing direct regeneration of genome-edited plants from somatic cells (Maher et al., 2020). The schematic representation of this process of gene delivery is given in **Figure 4B**.

##### 3.7.1.3 Cut-dip-budding method

A recently developed simple cut–dip–budding (CDB) delivery technique used *Agrobacterium rhizogene* to inoculate explants, resulting in altered roots that generate transformed buds as a result of root suckering **(** **Figure 4C**). The CDB method has achieved the heritable transformation of plant species in numerous plant families, including two herbaceous plants (*Taraxacum kok-saghyz* and *Coronilla varia*), a tuberous root plant (sweet potato), and three woody plant species (*Ailanthus altissima*, *Aralia elata*, and *Clerodendrum chinense*). Previously, it was difficult or impossible to change these plants, but the CDB approach permitted effective transformation or gene editing utilising a relatively simple explant dipping procedure, under non-sterile circumstances, without the requirement for tissue culture (Cao et al., 2023). This research implies that a huge number of plants might be genetically modified utilizing the CDB technique.

#### 3.7.2 Nanoparticle mediated gene delivery

**Figure 5:**
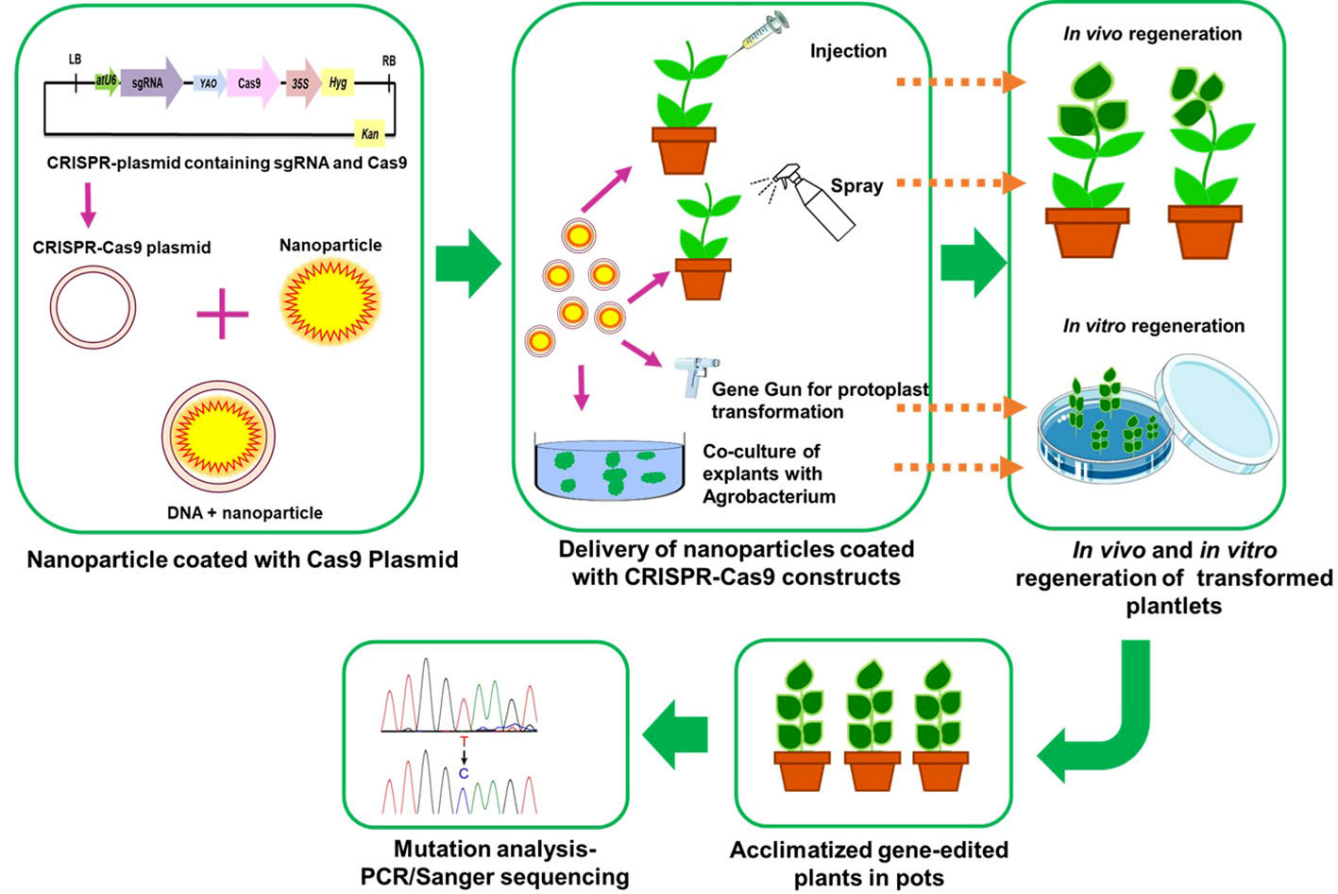
A schematic representation of nano-particle mediated delivery of CRISPR-Cas9 construct into plants.

Rapid progress has been made in nanoparticle-mediated gene transformation because it allows researchers to bypass the plant cell wall and enter the cell membrane. Recent studies have examined the use of nanomaterials including carbon nanotubes (CNTs), carbondots, mesosporous silicon nanoparticles (MSNs), clay nano sheets and DNA nanostructures to transport biomolecules such DNA, RNA, RNPs, and proteins (Mitter et al., 2017; Sandhya et al., 2020). Nanoparticles including DNA nanostructures have been used to successfully insert DNA and proteins into the nuclear and chloroplast genomes of plants (Kwak et al., 2019; Lv et al., 2020; Mujtaba et al., 2021; Laforest and Nadakuduti, 2022). Integrating multiple gRNAs, together with the appropriate promoters and terminators, into a single plant transformation vector allows for the regulation of different pathways. It will be challenging to introduce a big construct or a high number of gRNAs into plant cells due to their size. Therefore, nanoparticles and polycistronic tRNA-gRNA or polycistronic-Cys4-gRNA will be useful for multiple editing without the need of transgenes (Sandhya et al., 2020). It is important to supply CRISPR-Cas components to the plant protoplast, yet its low regeneration frequency might compromise editing efficiency. The scope and limitations of NP-mediated plant genetic engineering have been explored in recent works (Lv et al., 2020; Ghogare et al., 2021). Nanoparticles are advantageous for delivering gene editing components because they are not restricted to a protoplast but may instead be introduced into regenerative tissues of plant. However, the inefficiency of this approach might be attributed to the necessity for nanoparticles with a high carrying capacity of CRISPR-Cas components. Because genome editing technology is vital in crop improvement, combining gene editing with nanotechnology and *de novo* regeneration may speed up crop breeding (Lv et al., 2020; Gordon-Kamm et al., 2021). The schematic representation of nanoparticle mediated delivery in crops is given in **Figure 5**.

## 4 Genome editing using CRISPR-Cas system for consumer preferred trait improvement in crops

**Figure 6:**
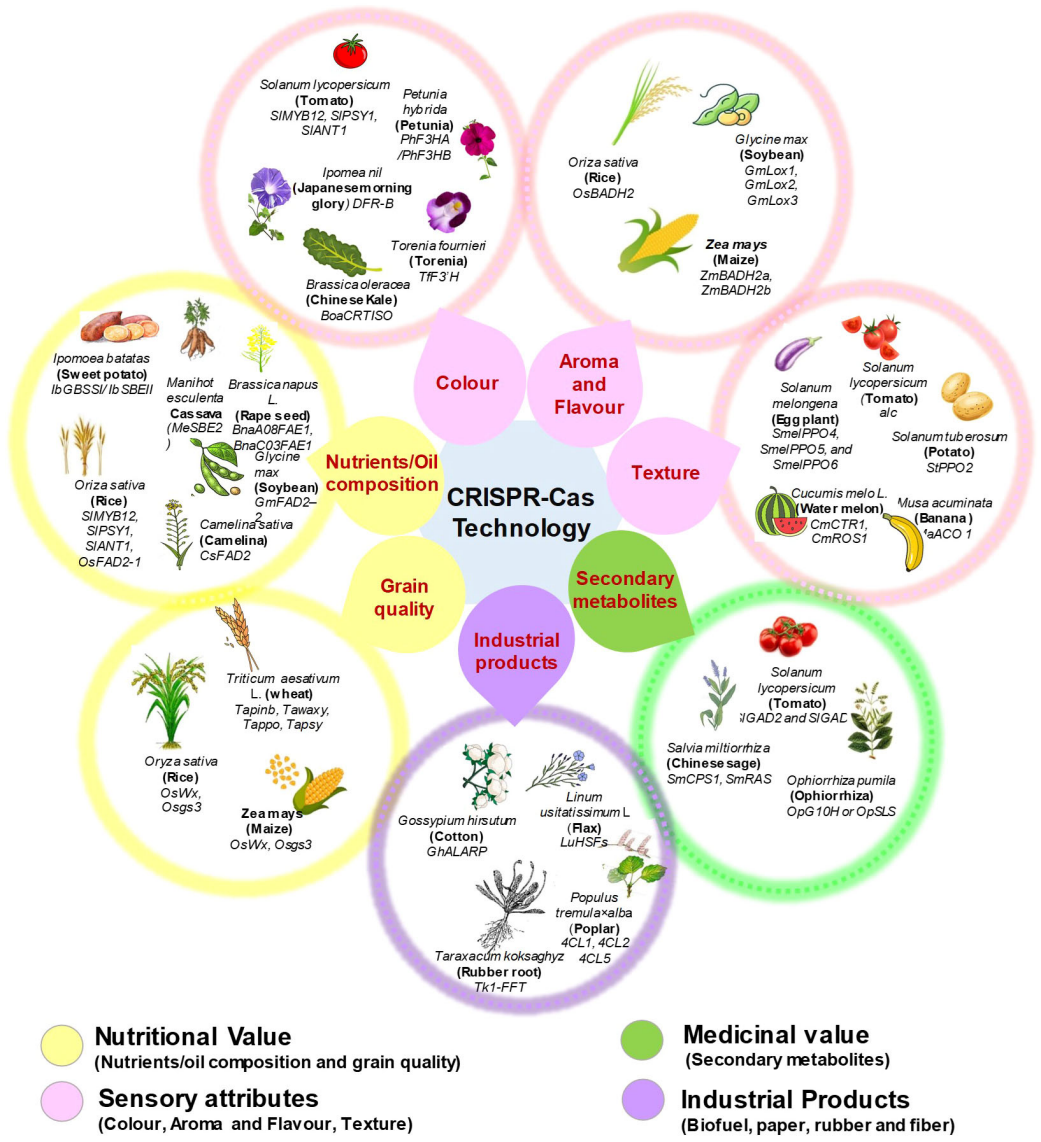
CRISPR-Cas technology based improvement of consumer preferred traits among various crops.

CRISPR-based technologies are hastening the identification of genes and characteristics in model or crop species by demonstrating the practicality of genome-wide and high throughput functional genomics (Meng et al., 2018). At the moment, crop gene editing is progressing faster than that in other areas. Even though some gene-edited crops, such as TALEN-*fad2* soybean, TALEN-*ppo* potato, and CRISPR-*wx1* maize, have been commercialized, this gene-editing revolution is still in its infancy. But, it is essential to realise the enormous potential of genome editing in plants by accelerate the process of creation of better crops with quality features (Oliva et al., 2019). Enhancing characteristics that are useful to individual customer is one of the primary objectives of crop research and development. Thus, we have summarized the applications of CRISPR-Cas technology in diverse crops species (cereal, fruit, vegetable, ornamentals and medicinal crops) for consumer specific commercial trait improvement **(** **Figure 6** and **Table 1** **).**

**Table 1:** Examples of the application of CRISPR-Cas technology in diverse crops species (cereal, fruit, vegetable, ornamentals, and medicinal crops) for consumer specific commercial trait improvement.

Crop name	Traits	Genes	Mutation type	Reference
*Solanum tuberosum*	High amylose content	*StSBE1, StSBE2*,	Base edition/deletion	Tuncel et al., 2019
	Anti-browning	*StPPO2*	Knock out	González et al, 2020
		*gbssI*	Knock out	Toinga-Villafuerte et al., 2022
*Ipomoea batatas*	High amylose content	*IbGBSSI, IbSBEII*	Base edition/deletion	Wang et al., 2019
*Solanum lycopersicum*	High GABA content	*SlGAD2, SlGAD3*	Base edition/deletion	Nonaka et al., 2017
	Increased lycopene content	*slyPDS, BnFAD2*	Base edition/deletion	Li et al., 2018
	Increased lycopene content	*SlSGR1, SlBlc*	Base edition/deletion	Li et al, 2018
*Ophiorrhiza pumila*	Secondary metabolite ehancement	*OpG10H or OpSLS*	Base deletion	Shi et al., 2020
*Solanum melongena*	Polyphenol content	*SmelPPO4, SmelPPO5, and SmelPPO6*	knock-out	Maioli et al., 2020
*Ipomea nil*	Colour	*DFR-B*	Base edition/deletion	Watanabe et al., 2017
*Brassica napus*	High oleic acid proportion	*BnFAD2*	Base deletion	Liu et al., 2022
	Low phytic acid content	*BnITPK*	Base edition/deletion	Sashidhar et al., 2020
	High oil production and GPC	*BnTT8*	Base edition/deletion	Zhai et al., 2020
	Increased in size of the oil bodies	*Bnlpat2, Bnlpat5*	Base edition/deletion	Zhang et al., 2019
	Lignin contents	*BnSHP1, BnSHP2*	Base edition/deletion	Zaman et al., 2021
	Oil content	*BnaA08FAE1, BnaC03FAE1*	Nucleotides deletion	Liu et al., 2022
*Artemisia annua*	Artemisin	*Squalene synthase (SQS)*	Nucleotides deletion	Koerniati and Simanjuntak, 2020
*Torenia fournieri*	Color	*TfF3H*	Base edition/deletion	Nishihara et al., 2018
*Oriza sativa*	Aroma	*OsBADH2*	Nucleotides deletion	Ashokkumar et al., 2020
	Monounsaturated fatty acids synthesis	*OsFAD2-1*	Base deletion	Bahariah et al., 2021
	Lignin content	*Os4CL3 and Os4CL4*	Knock out	Afifi et al., 2022
*Gossypium hirsutum L.*	Fiber development	*GhMYB25-like A*, *GhMYB25-like D*	Base edition/deletion	Li et al., 2017
*Gossypium hirsutum* (Cotton)	Fiber development	*GhALARP*	Base deletion	Zhu et al., 2018
*Populus tremula×alba*	Lignin biosynthesis	*4CL1, 4CL2* *4CL5*	Knock out	Tsai et al., 2020
*Zea mays*	Aroma	*ZmBADH2a, ZmBADH2b*	Knock out	Wang et al., 2021
*Taraxacum kok-saghyz*	Rubber synthesis	*1-FFT*	Base edition/deletion	Iaffaldano et al, 2016
*Glycine max*	Oleic acid	*FAD2–2*	Base edition/deletion	Aman et al., 2018
	Flavor	*GmLox1, GmLox2, GmLox3*	Base deletion	Wang et al., 2020
*Camelina sativa*	Monounsaturated Fatty Acid	*FAD2*	Base edition	Lee et al., 2021
*Cucumis melo*	Fruit ripening	*CmCTR1, CmROS1*	Base deletion	Giordano et al., 2022
*Arachis hypogaea*	High oleic acid oil content	*FAD2*	Base edition/deletion	Neelakandan et al., 2022
*Medicago truncatula*	Triterpene saponins	*CYP93E2 and CYP72A61*	Base deletion	Confalonieri et al., 2021
*Salvia miltiorrhiza*	Diterpene enhancement	*SmCPS1, SmRAS*	Base deletion	Li et al., 2017
	Lignin formation	*SmLACs*	Base deletion	Zhou et al., 2021
*Atropa belladonna*	Tropane alkaloid biosynthesis	*PYKS*	Knock out	Hasebe et al., 2021

### 4.1 Grain quality/nutritional content/oil composition

Recent consumer trends reflect a greater appreciation for the nutritional value of food grain, fruits and vegetables. As economic growth continues, the demand for premium grain quality increases. In wheat, four grain quality related genes were targeted by genome editing: *pinb, waxy, ppo, and psy*, which are involved in wheat grain hardiness, starch and dough colour, respectively. As a result, novel wheat germplasms with enhanced grain quality in terms of hardness, starch content, and dough colour were produced (Zhang et al., 2021). In rice (*Oryza sativa* L.), the diverse actions of natural *Wx* alleles govern distinct amylose contents (AC), gel consistency (GC), and pasting viscosity of grain starches; these parameters together affect grain appearance, cooking/eating quality, and starch physical properties (Zhang et al., 2019). Quantitative modulation of Waxy allele expression by CRISPR-Cas9-based promoter and 5’UTR-intron editing increases rice grain quality and the quality of rice products to satisfy customer’s preference (Zeng et al., 2020). Grain size is one of the primary characteristics that impact the productivity and quality of rice grains. The *GS3* gene is the first significant quantitative trait locus (QTL) found to regulate the length and weight of rice grains. CRISPR-Cas9 gene-editing technology was used to introduce an altered *gs3* allele into the indica maintenance line *Mei1B* in order to improve its grain production and quality (Huang et al., 2022). Luo et al. (2022) were the first to produce high-amylose cassava by CRISPR-Cas9-mediated mutation of the starch branching enzyme gene *SBE2*. Thus, CRISPR technology was proved to be a successful method for creating new starch types for food and commercial purposes. Cereals, high in resistant starch (RS) may be beneficial to health and might help to protect protect against chronic diseases that are linked to poor nutrition. Multiplex CRISPR–Cas9 gene editing of starch branching enzyme genes boosted ‘Presidio’ rice’s resistant starch. With the help of agrobacterium-mediated transformation, the CRISPR-Cas9 vector construct containing four sgRNAs targeting the *SBE* gene was introduced into the American rice cultivar Presidio. Eight transgene-positive T0 plants had all four *SBE* gene knockout variants. SBE-edited lines have up to 15% more RS than the wild-type (WT) cultivar Presidio (Biswas et al., 2022). Mutagenesis of starch biosynthetic genes in sweet potatoes (*Ipomoea batatas*) using CRISPR-Cas9 was used to enhance starch quality (Wang et al., 2019).

Enhancement of nutritional content and reduction of anti-nutrition factors in food crops is among preferred consumer’s trait. Non-proteinogenic amino acid -aminobutyric acid (GABA) has been shown to reduce blood pressure in hypertensive individuals who consume it orally. An increase in GABA levels may further improve the blood pressure reducing effect of tomato fruit. Using CRISPR-Cas9 technology, researchers knocked out an autoinhibitory domain of Glutamate decarboxylase (*GAD*) genes (a crucial enzyme in GABA production) such as *SlGAD2* and *SlGAD3* in tomato to boost its GABA content (Nonaka et al., 2017). Oils extracted from seeds have both culinary and industrial uses. In contrast to monounsaturated fatty acids (MUFA) most seed oils are high in polyunsaturated fatty acids (PUFAs). Soybeans had their *FAD2-2* microsomal omega-6 desaturase specifically disrupted using the CRISPR-Cas9 nuclease technology (*Glycine max*.L). The oleic acid content of the edited lines varied significantly, reaching a maximum of 65.58% whereas the linoleic acid concentration reached a minimum of 16.08% (Al Amin et al., 2019). Three homologs of the gene for fatty acid desaturase 2 (*FAD2*), the enzyme that converts oleic acid into linoleic acid, have been found in the genome of the new oilseed crop camelina. Knockout plants lacking the *CsFAD2* gene were created using CRISPR-Cas9 to boost the MUFA content of Camelina seed oil (Lee et al., 2021). Improvement of the fatty acid composition in Rice bran oil (RBO), *OsFAD2-1* gene was disrupted by CRISPR-Cas9-mediated targeted mutagenesis. Homozygous *OsFAD2-1* knockout mutant rice plants have increased oleic acid compared to wild type (Abe et al., 2018). The breeding of soybean to lower its saturated fatty acid (FA) content, which has been related to cardiovascular disease, would have a significant impact on nutritional enhancement. Obtaining CRISPR-Cas9 mutants that lack the *GmFATB1* (Acyl-acyl carrier protein thioesterases) gene resulted in a shift in oil profile, indicating that there is considerable potential for increasing the quality of soybean oil for human health (Ma et al., 2021). The processing and nutritional value of vegetable oils might be negatively impacted by the presence of erucic acid (EA). That’s why brassica with low EA has been a desirable feature for generations. The enzyme fatty acid elongase 1 (*FAE1*) plays a crucial role in the production of EA. Three *Brassica napus* germplasms (high EA (>30%) and oil (>50%)) were mutated specifically in two homologous copies of *BnaFAE1* utilising CRISPR technology. In *BnaFAE1*-edited germplasms, EA content was dramatically decreased, whereas oleic acid content was elevated to varying degrees (Liu et al., 2022).

### 4.2 Sensory attributes

Consumer acceptance of a crop is determined by factors other than its nutritional content, such as sensory qualities (i.e., colour, texture, and flavour). Appearance is the primary determinant of consumer preference, whereas other aspects maintain future purchases. The market for horticultural crops, such as fruits, vegetables, and floricultural crops, places a premium on such sensory qualities. Diverse crops or their harvests provide a spectrum of hues (e.g., green, yellow, red, purple, or multicolour), flavours (e.g., neutral, slightly sour, spicy, or bitter), and textures. Among horticulture/ornamental crops, visual aspect and fragrance are important elements for overall plant quality and novel flower colour production is regarded as one of the chief commercial demand of consumers. Other sensory characteristics, such as aroma and texture/vase life, are also highly prized for their aesthetic and economic potential. Therefore, generation of crop varieties with desired colour, improved fragrance, and rich texture using gene editing technology may become prospective benefit to both growers and consumers.

#### 4.2.1 Aroma and flavor

Aroma and flavour are important sensory attributes that influences product’s acceptance and marketability. In both Asia and Europe, rice-eating populations favour fragrant rice varieties. Multiple plant species beside rice contain a naturally fragrant germplasm, all of which have a reduced level of BETAINE ALDEHYDE DEHYDROGENASE 2 activity (BADH2). Targeted mutation using CRISPR technology of rice *OsBADH2* gene generates novel alleles of *OsBADH2*, resulting in fragrance production in non-aromatic rice types (Ashokkumar et al., 2020). The use of CRISPR-Cas to create a double *zmbadh2a-zmbadh2b* mutant in maize resulted in popcorn-like odour in the seeds of the double mutant. These results reveal that *Zmbadh2a* and *Zmbadh2b* participate in 2AP production in maize in a redundant manner and suggest to the development of the world’s first aromatic maize by simultaneous editing of the two *BADH2* genes (Wang et al., 2021). Beany flavour caused by three lipoxygenases (*LOXs*) (*LOX1, LOX2*, and *LOX3*) limits human intake of soybean. To increase the eating quality of soybean oil and protein products, it is preferable to produce lipoxygenase-free novel mutant lines. Three *GmLOX* genes (*GmLox1, GmLox2, and GmLox3*) were altered using CRISPR-Cas9 method. Plants with various combinations of mutations lost the associated lipoxygenase activity. These lipoxygenase-deficient mutants might be utilised to reduce the beany taste of soybeans (Wang et al., 2020).

#### 4.2.2 Color

Horticulturists/floriculturists and related entrepreneurs are always eager to obtain novel colours in flower, fruit and vegetables. Usually, sensory characteristics like colour and the chemical composition of a product are intertwined. As most of the pigments such as carotenoids, anthocyanins, and polyphenols determine plant colour. The colour of plant edible parts, particularly fruit, leaves, and flower buds, influences customer choice. Tomatoes are one of the most significant industrial crops, and they are frequently employed in the production of industrial food items. The pigment and colour of tomatoes are key commercial traits. Manipulation of fruit colour may therefore be accomplished by interrupting genes involved in the pigment production pathway using CRISPR-Cas9. *SlMYB12*, an *R2R3-MYB* transcription factor plays a crucial function in flavonoid accumulation. The *SlMYB12* gene was knocked out effectively using CRISPR-Cas9 mediated mutagenesis, resulting in pink tomato fruit (Deng et al., 2018; Yang et al., 2019). Targeting *PSY1* and *ANT1*, the researchers produced yellow and purple tomatoes. The enzyme phytoene synthase is encoded by the *PSY1* gene, which controls the first phases of the carotenogenesis process. Tomatoes with yellow flesh resulted from *PSY1* mutations that drastically decreased overall lycopene concentration (Filler Hayut et al., 2017), whereas *ANT1*-edited tomatoes increased anthocyanin accumulation and yielded purple plant tissue (Vu et al., 2020). The edible organs of most kinds of Chinese kale are green, with the exception of a few cultivars with red bolting stems. To modify the hue and pigment concentrations of Chinese kale, the carotenoid isomerase gene (*BoaCRTISO*) was edited by employing the CRISPR-Cas9 system. The homozygous mutants transformed from green to yellow, most likely as a result of a decrease in the color-masking action of chlorophyll on carotenoids (Sun et al., 2017). In another study, CRISPR-Cas9 genome editing was employed to independently knock out *BoDFR1* or *BoDFR2* in the pink-leaved ornamental kale. Mutation in *Bodfr1* resulted in very low accumulation of anthocyanins (Zhang et al., 2022). In all of the crop species investigated, *R2R3-MYB, bHLH*, and *WD-*repeat proteins play major roles in regulating the anthocyanin biosynthesis structural genes. *DcMYB7*, and *R2R3-MYB*, was knocked out in the solid purple carrot using CRISPR-Cas9, resulting in yellow roots (Xu et al., 2019). Plant breeders are continually looking for new colours in flowers as it is one of most prominent characteristic of fresh flowers which presumably plays a significant effect in consumer preference and influences market value of ornamental crops. Flavanone 3-hydroxylase (*F3’H*) is required for anthocyanin accumulation since it is a crucial enzyme in flavonoid production. Light blue torenia flower variants and pale purplish-pink petunia flower varieties are the results of CRISPR-Cas9-mediated disruption of F3’H (Nishihara et al., 2018; Yu et al., 2021).

#### 4.2.3 Texture

Enzymatic browning is an issue with few crops such as pototo and eggplant that occurs during harvest and post-harvest processing, resulting in nutritional quality, taste, and texture loss. Browning of potato tuber has been minimised by CRISPR-Cas9 ribonucleoprotein system disruption of a polyphenol oxidase (PPO) gene. This approach has shown that mutations in the *StPPO2* gene reduce Tuber PPO activity up to 68% and 73%, respectively (González et al, 2020). Ten *PPO* genes (SmelPPO1-10) were identified in *Solanum melongena* L. when a high-quality genome sequence became available recently. By utilizing a CRISPR-Cas9-basedmethod three target *PPO* genes (*SmelPPO4*, *SmelPPO5*, and *SmelPPO6*) were knocked out. The induced mutations were inherited stably in the T1 and T2 offspring and were associated with reduced PPO activity and browning of the berry flesh upon cutting. Because of this, it’s likely that genotypes with little browning of the flesh and high polyphenol content in the berries will become established (Maioli et al., 2020). Because of the presence of climacteric and non-climacteric types, melon (*Cucumis melo* L.) has arisen as an alternative model for studying fruit ripening. To understand the role of *CmCTR1*-like and *CmROS1* genes in climacteric ripening, homozygous CRISPR knockout mutants of these genes were generated in a climacteric genetic background. In two summer seasons, both loss-of-function mutants showed accelerated ethylene production relative to the climacteric wild type, demonstrating a role for both genes in melon climacteric ripening (Giordano et al., 2022). As a typical climacteric fruit, banana will ripen and decay in one week after exogenous ethylene induction. In climacteric fruit, the transcription of 1aminocyclopropane1carboxylic acid (*ACC*) synthase (ACS) and ACC oxidase (*ACO*) genes regulates ethylene production. ACO is responsible for the reduction process that converts ACC to ethylene. Bananas keep up a good texture for longer period of time under natural ripening conditions, after having their *MaACO1* (aminocyclopropane1carboxylate oxidase 1) gene edited using CRISPR-Cas9 (Hu et al., 2021)

### 4.3 Secondary metabolite and phytopharmaceuticals

It is generally well known that the various secondary metabolites of medicinal plants have therapeutic capabilities. Gene editing holds potential for enhancing the consumer traits with regard to the production of medicines and therapeutics from medicinal plant species. *Salvia miltiorrhiza*, a medicinal plant, has been effectively edited by employing CRISPR-Cas9 driven knock out of the diterpene synthase gene (*SmCPS1*) involved in tanshinone production without interfering with other phenolic compounds (Li et al., 2017). This will undoubtedly open the way for large-scale genome editing in *S. miltiorrhiza*, with the goal of elucidating the process for secondary metabolite production, improving quality, and increasing yields of this important traditional Chinese medicinal plant. Camptothecin (CPT) is a natural chemical with outstanding anticancer effects. *Ophiorrhiza pumila* produced camptothecin, its concentration in hairy root lines mediated by CRISPR-Cas9 system (*OpG10H or OpSLS* knock-out lines) was clearly decreased, showing that both *OpG10H* and *OpSLS* play crucial roles in CPT biosynthesis. Laccases, which are involved in the synthesis of significant medicinal phenolic acid compounds like salvianolic acid B (SAB), used to treat cardiovascular disease, are another attractive target for gene editing in salvia. Multiple laccase genes in salvia were targeted using the CRISPR-Cas9 technology, leading to a significant reduction in target laccase gene expression and phenolic acid in gene edited lines. There is a significant function for rosmarinic acid, a kind of aqueous phenolic compounds, in the treatment of inflammatory illnesses. Improved rosmarinic acid quality has resulted from the use of CRISPR to silence the rosmarinic acid synthase (*SmRAS*) gene in salvia. This has enhanced synthesis of 3, 4-dihydroxy phenyl lactic acid (Zhou et al., 2018).

### 4.4 Industrial products: biofuel, paper, rubber and fiber

The reduction in lignin content and modification of its composition in cell wall of plants increase the appropriateness of lignocellulosic biomass for the pulp, paper, and textile industries, biofuel and feed (Verma and Dwivedi, 2014; Capstaff and Miller, 2018). Genetic modification can reduce lignin content and enhance saccharification efficiency, but usually at the cost of moderate-to-severe growth penalties. A single DNA construct was employed in CRISPR-Cas9 gene editing to knock out expression of an endogenous gene of lignin monomer biosynthesis while simultaneously expressing a modified version of the gene’s open reading frame that escapes cleavage by the Cas9 system and complements the mutation in a tissue-specific manner. By expressing the complementary open reading frame in vessels, it is possible to regenerate arabidopsis plants with less lignin, wild-type biomass production, and up to a fourfold increase in cell wall sugar yield per plant (Yu et al., 2021). It has been reported that the CRISPR-Cas9 system was used to target two *4CL* (4-coumarate: CoA ligase) genes, *4CL1* and *4CL2*, involved in lignin and flavonoid biosynthesis, respectively, in the woody perennial Populus, with 100% mutation efficiency for both genes and biallelic modifications in every transformant tested (Zhou et al., 2015). CRISPR-Cas9-mediated mutation of *HvCOMT1*, the lignin biosynthetic gene that forms lignin syringyl units, reduces barley’s lignin content (Lee et al., 2021). Caffeoyl shikimate esterase (*CSE*), which helps plant to synthesize lignin, is a prospective target for improving lignocellulosic biomass crops for sustainable biofuel generation. CRISPR-Cas9-mediated knockouts of each *CSE* gene in transgenic hybrid poplars improved saccharification efficiency by reducing lignin content (Jang et al., 2021). Natural rubber (NR) is an important raw material for a large number of industrial products. The primary source of NR is the rubber tree *Hevea brasiliensis*, but increased worldwide demand means that alternative sustainable sources are urgently required. As a natural rubber substitute, the roots of the *Taraxacum kok-saghyz* (TK-Rubber Dandelion) plant generate a high-molecular-weight rubber. As inulin is thought to be counterproductive to rubber production, the domestication of TK was hastened by targeting the fructan:fructan 1-fructosyltransferase (*1-FFT*) gene, which encodes an enzyme involved in inulin manufacture, using the CRISPR-Cas9 gene-editing tool (Iaffaldano et al., 2016). *Linum usitatissimum* L., a worldwide cash crop, produces linseed oil and linen. High temperature stress limits flax growth. The studies of Saha et al. (2019) identified 34 flax genome-wide HSF genes and created guide RNA sequences for gene editing with minimal off-target consequences. Genome engineering *LuHSFs* will create high-temperature stress-tolerant flax cultivars and boost fibre output. Using the CRISPR-Cas9 technology, targeted mutations in *GhALARP* (a gene primarily expressed in cotton fibres that encodes an alanine-rich protein) have been produced (Zhu et al., 2018). These mutants provided evidence for deducing the role of *GhALARP* in the formation of cotton fibre.

## 5 Challenges and future directions

Now a days, genome engineering through CRISPR has become a powerful and efficient tool for targeted gene modifications. Although genome editing offers numerous benefits over traditional crop breeding, still certain challenges remained unsolved before it can be applied to improve various commercially important consumer specific traits in crops.

### 5.1 Organelle genome editing

Mitochondria and chloroplasts, having their individual genomes that encode many genes required for biological processes such as respiration and photosynthesis, respectively. The lack of efficient ways for targeting DNA in plant organelles has hampered plant organelle gene editing, which is an unmet need in plant biology fundamental research (Kang et al., 2021). Methods for editing these genes in organelles are in great demand for elucidating their functions and improving vital traits. For instance, targeted mutagenesis in the mitochondrial *atp6* gene may result in male sterility (Howad and Kempken, 1997), which is a beneficial trait for crop breeding, while a precise point mutation in the *16S rRNA* gene of the chloroplast genome results in antibiotic resistance (Fromm et al., 1989). For crop improvement, CRISPR-Cas has been utilized to edit nuclear genomes. These techniques have never been used to target higher plant plastid genomes. Despite the fact that there are no nuclease-mediated genome editing approaches for plastome engineering, homologous recombination-based plastid transformation may introduce point mutations (Bock, 2015). The biggest hurdle in using CRISPR-Cas mediated mtDNA and cpDNA editing is introducing both sgRNA and Cas9 endonuclease into these double-membraned organelles (Glass et al., 2018). Even though, mitochondrial genome editing with CRISPR-Cas9 have been reported in human cells, but the results aren’t clear (Yang et al., 2021a). Optimising CRISPR-Cas system organelle genome editing in multicellular plants will definitely make it a very useful tool for crop improvement programme. To improve CRISPR-Cas9 system for editing mitochondrial DNA a modified sgRNAs that can target mitochondria should be used. This is done by adding the RNA transport-derived stem loop element (RP-loop) to the sgRNA, which lets it transport into mitochondria. This resulted into the reduced transcript levels of the targeted genes of mtDNA in human cells and revealed that sgRNA can introduced into organelles by using the native machinery for introducing RNA into organelles (Hussain and Kumar, 2021). But the ways that organisms bring RNA into organelles are very different and are mostly unknown in plants (Tian et al., 2018). Gómez and Pallas (2012) found that addition of an internal sequence of 110bp from the eggplant latent viroid to the 5′ end of the green fluorescent protein (*GFP*) mRNA caused this chimeric RNA to be imported into the chloroplasts of *Nicotiana benthamiana*. Recently Kang et al. (2021) used DddA-derived cytosine base editor (DdCBE)2 to induce point mutations in mitochondria and chloroplast DNA. This DdCBEs triggered base editing at rates of up to 25% (mitochondria) and 38% (chromosomes) in lettuce or rapeseed calli (chloroplasts). In order to prevent off-target mutations produced by DdCBE-encoding plasmids, researchers have also demonstrated DNA-free base editing in chloroplasts by delivering DdCBE mRNA to lettuce protoplasts. Using bacterial cytidine deaminase linked to the DNA-binding domains of transcription activator-like effector nucleases, targeted base editing in the *Arabidopsis thaliana* plastid genome has been accomplished. In certain plantlets of the T1 generation, the targeted Cs were homoplasmically swapped with Ts, and the mutations were acquired by their progeny regardless of the nuclear vectors delivered (Nakazato et al., 2022). To improve consumer specific traits like medicinal/industrial value in crops, editing of organelle genome is crucial as chloroplast genome harbors various biosynthetic pathways, including the shikimate, *de novo* fatty acid synthesis, and methylerythritol 4-phosphate pathways which serves as a precursor for a wide range of commercially important secondary metabolites, including tocopherols, pigments, and many phytohormones (Li et al., 2021). Still, few cues remain a challenge in organelles both chloroplast and mitochondria genome transformation such as having a double membrane, poor gene expression in non-green plastids and the limited host range of agrobacterium.

### 5.2 Ploidy level

Polyploidy is the acquisition of one or more extra full chromosomal sets inside an organism. There are at least three distinct types of polyploidy: autopolyploids, allopolyploids, and segmental allopolyploids. Due to their huge genome size and repetitive sequences, polyploids genomes are challenging to sequence. Because many alleles must be edited concurrently, gene knockout effectiveness is often lower in polyploid plant species than in diploids (Ahn et al., 2020). An effective expression system and a highly active Cas nuclease are essential for successful polyploid plant genome editing. Multiallelic genome editing has been accomplished in various polyploid plant species, including both model systems and crop species till date (Weeks, 2017; Naim et al., 2018; González et al., 2020; Lin et al., 2020). In tetraploid potato, CRISPR-Cas9 base and prime editors have been used to alter the catalytic motifs of the *GBSSI* (granule-bound starch synthase) gene (Toinga-Villafuerte et al, 2022). Using CRISPR-Cas9 RNPs to induce mutations in the polyphenol oxidase 2 gene (*StPPO2*) in tetraploid potato led to a significant decrease in enzymatic browning following cutting (González et al, 2020). Earlier, genome editing through CRISPR-Cas9 for the improvement of grain quality (important trait for consumers) including grain and kernel weight and storability in hexaploid wheat have been studied by Dayani et al. (2019). PolysgRNAs tRNA-mediated genome editing may be used to specifically target the existence of multiple copy numbers in polyploidy crops. Furthermore, environmental stress, epigenetic alterations, physiological and cellular responses to stress contribute to a rise in polyploidy (Van de Peer et al., 2021). In an octaploid crop like strawberry, fruit colour affects consumer preference and is an important trait for breeding. Using CRISPR-Cas9 technology, Gao et al. (2020) knock out six of eight copies of the Reduced Anthocyanins in Petioles (*RAP*) gene in cultivated octaploid strawberry plants, resulting in altered fruit coloration. Multisite alterations in the genome are possible using CRISPR-Cas. In contrast to diploid crops, where building a CRISPR system is relatively straightforward, polyploid crops provide more of a challenge owing to the availability of many alleles of target genes. Moreover, accurate genome editing using HDR in stable transgenic lines of polyploid plants, in addition to gene knockdown, remains difficult. Thus to achieve mutations in all different alleles in polyploid crops, the design of guide RNAs require special attention. The simultaneous editing of numerous homologs using CRISPR-Cas technology without any mutations in the background would provide novel breeding opportunities for mutant genotypes.

### 5.3 Off-targeting

Off targeting involves cleavage and genomic DNA modification outside the target region. Off targeting is a critical challenge when using CRISPR-Cas system to edit genes associated with traits (Wolt et al., 2016). Cas9-induced double-strand breaks may lead to significant deletions and genomic rearrangements. Several methods are available to detect the off-targets which includes deep sequencing, web-based prediction tools, FISH, HTGTS, IDLV, Digenome- seq, T7E1 assay, Guide -seq, and chip-seq (Cho et al., 2013; Ran et al., 2013; Heigwer et al., 2014; Tsai et al., 2015; Paulis et al., 2015). In plants, much research has been conducted to discover off-target impacts sequencing the whole genome of arabidopsis, rice, cotton, etc. Studies have revealed that genome editing in plants using wild-type Cas9 and Cas12a has remarkable specificity, revealing that the majority of mutations discovered in edited plants are the consequence of somaclonal alterations (Swarts and Jinek, 2018). CBEs, produce genome-wide off-target effects in rice, as proven by whole genome sequencing; hence, their usage may require extra screening and purifying selection (Doman et al., 2020). However, base editors may be designed to restrict their RNA editing activity significantly. To reduce the likelihood of off-target mutations, paired nCas9s might be utilized. High fidelity versions of SpCas9 have been developed *via* protein engineering to lower Cas binding affinity (and hence increase editing specificity). In rice, the on-target editing activity of eSpCas9 (versions 1.0 and 1.1) and SpCas9-HF1 is preserved, while the specificity is improved by using the t-RNA-sgRNA processing system (Huang et al., 2022). Two versions of Cas9, eHF1-Cas9 and eHypa-Cas9, have been shown to successfully alter the rice genome. Recent research indicates that xCas9 has greater targeting selectivity than wild-type Cas9 in rice (Zhang et al., 2019). However, due to the inherently reduced nuclease activity of many high-fidelity SpCas9s in plants, their use for plant genome editing is less reliable until additional advancements are made. Other strategies for minimising off-target effects include the development of gRNAs with fewer possible mismatch targets in a given genome. In addition, restricting the genome’s exposure to CRISPR reagents, such as through transient expression and RNP transformation, may lower the likelihood of off-target activity (Xu et al., 2019).

In terms of precision and efficiency, mutagenesis by CRISPR-Cas9 mediated genome editing outperforms spontaneous and induced mutations. Organelle genome editing, higher ploidy levels, complex genome/lack of genome sequence, and off targeting are important hurdles in improvement of commercially viable/consumer preferred traits in crops. But given the immense potential of genome editing, we anticipate that these problems will be solved in the near future.

## 6 Safety regulations and considerations

Genome editing is one of the new generation breeding tools used by public and private breeders to develop new crop varieties (Gleim et al., 2020). Although there are few arguments raised related to global biosafety regulations and social concerns about the use of CRISPR-Cas9 tools in plants (Endo et al., 2019). Off targeting is one of the major concerns creating unwanted genetic changes in the plants. Second the social concerns related to CRISPR-Cas9 is lack of information about its principal, application and distinction between genetically modified plants and genome edited plants (Eckerstorfer et al., 2019). Several ethical considerations prevent genome editing from being widely used for crop improvement since its results are not significantly different from those acquired from natural spontaneous or induced mutations. The vast majority of nations are re-evaluating and adjusting their biosafety laws and rules to accommodate this adaptable and useful technology. The European Union (EU) and New Zealand are two examples of countries that classify genome-edited crops as genetically modified organisms (GMOs), and hence regulate them as such (Friedrichs et al., 2019). In nations like as Argentina, Australia, Brazil, Canada, Chile, Japan, and the United States, the ultimate result of genome editing determines whether a crop is classified as GM or not (Van Vu et al., 2019). The stances of the vast majority of nations have not yet been defined; nevertheless, a few of countries, like Nigeria and Kenya, have begun formulating regulatory guidelines for genome-edited crops (Eckerstorfer et al., 2019; Tripathi et al., 2020). Legally, gene edited plants in China are considered GMOs, although detailed regulations for genome-edited crops have not been released (Gao et al., 2018). The United States Department of Agriculture approved *Camelina sativa* (fake flax) with increased omega-3 oil that had been gene edited. Furthermore, in the United States, regulations do not apply to the CRISPR edited drought-resistant soybean variety. India also exempted gene-edited crops from bio-safety evaluation in 2022 and confirmed that SDN1 and SDN2 variants contain no alien DNA and can be treated as conventional hybrids (Brock et al., 2022). By inactivating the *Wx1* gene, scientists have created waxy maize that is high in amylopectin have been exempted from GMO regulation (Nerkar et al., 2022). In another crop, *Setaria viridis* edited with a homolog of the maize *ID1* gene to delay flowering will be exempted from USDA control (Taylor et al., 2020). A white button mushroom (*Agaricus bisporus*) that is resistant to browing has been exempted from regulation policies (Waltz, 2018). In Canada, Cibus Canada Inc developed herbicide resistant canola by introducing single nucleotide directed mutagenesis in two genes using ODM (an oligonucleotide-directed mutagenesis) similar as CRISPR-Cas9. According to the Canadian government in 2013, the new canola variety was not distinguishable from traditional canola types and was classified as a non-GM crop by the Canadian Food Inspection Agency (CFIA, 2013). Globally, the path of genome editing regulation is not yet apparent. Different nations have different regulations for genome edited crops, resulting in an uneven worldwide regulatory structure that impedes the global use of genome-edited crops. As a result, there is a need to build a more realistic, optimistic, and universal regulatory framework on genome editing crops internationally, which will aid in bringing the globe under one regulatory regime.

## 7 Conclusion

CRISPR-Cas systems are being used in many aspects of crop improvement, in addition to basic research in plant sciences. This technology has tremendous potential for creating precise and targeted genetic variability in crops according to the need of farmers and consumers. But still, transformation and tissue culture protocol, lack of genomic resources remain bottlenecks in improvement of commercial traits through gene editing approach. The development of the new omics technology provides information about important target genes and their delivery through advanced methods will increase the prospects of this innovative technology for the improvements of consumer preferred traits in various crops. Public scientific seminars or workshops explaining the need and advantages of gene editing are necessary to address the ethical concerns that have been generated by its use. There are ongoing attempts to enhance editing capabilities and to comprehend the repercussions of genome editing as we enter this new technological age. Prior to submitting trials, it is necessary to do a molecular characterization of crops produced using genome-editing technology. To expedite the adoption of genome-edited crops in crop breeding, it is necessary to establish a pragmatic, product-based, global regulatory policy.

## Author contribution

VV: Conceptualization, writing-original manuscript and figures preparation, writing review and editing. AK: writing- original manuscript, table and figures preparation. MP: writing review, figures preparation, and editing. MT: writing review, figures preparation, and editing. BB: conceived the concept, data curation, visualization, writing-review and editing, supervision, funding acquisition, project administration. All authors contributed to the article and approved the submitted version.
